# Prior irinotecan exposure does not preclude benefit to liposomal irinotecan in patients with metastatic pancreatic ductal adenocarcinoma

**DOI:** 10.1002/cam4.5714

**Published:** 2023-03-19

**Authors:** Kenneth H. Yu, Paul Cockrum, Andy Surinach, Neil Lamarre, Shu Wang, Eileen M. O'Reilly

**Affiliations:** ^1^ Memorial Sloan Kettering Cancer Center and Weill Cornell Medical College New York New York USA; ^2^ Ipsen Cambridge Massachusetts USA; ^3^ Genesis Research Jersey New Jersey USA

**Keywords:** antineoplastic agents, electronic health records, overall survival, pancreatic ductal adenocarcinoma, real‐world evidence, treatment options

## Abstract

**Background:**

Subgroup analyses of the NAPOLI‐1 study identified that among patients who were irinotecan naïve prior to entering the clinical trial, a survival benefit was observed between the study arm and control arm. This treatment benefit was not observed among those previously exposed to irinotecan. This study sought to understand the impact of prior exposure to irinotecan on clinical outcomes among patients treated with liposomal irinotecan in the real‐world setting.

**Methods:**

This retrospective observational study utilized a nationwide electronic health record (EHR)‐derived deidentified database. Data for adult patients with mPDAC treated with liposomal irinotecan‐based regimens between January 2016 and October 2020 were analyzed. Patient characteristics, overall survival (OS), and progression‐free survival (PFS) were assessed. Cox proportional hazard methods were used to calculate hazard ratios (HRs). HRs were adjusted for demographics and relevant clinical covariates.

**Results:**

Six hundred and seventy‐five patients with mPDAC treated with a liposomal irinotecan‐based regimen were included. The unadjusted OS HR was 1.3 (95% CI: 1.1–1.6, *p* < 0.001) and unadjusted PFS was HR 1.4 (95% CI: 1.2–1.7, *p* < 0.001). After adjustment for baseline characteristics, the adjusted OS HR was 1.0 (95% CI: 0.8–1.3, *p* = 0.8836) and the adjusted PFS HR was 1.1 (95% CI: 0.8–1.4, *p* = 0.5626).

**Conclusions:**

Prior irinotecan was not found to be a significant predictor of patient outcomes in those later treated with liposomal irinotecan. Thus, the results may inform the rationale for utilizing liposomal irinotecan combination therapy following prior irinotecan exposure in mPDAC, in particular where the prior irinotecan exposure was more distant in time.

## 1 INTRODUCTION

Pancreatic ductal adenocarcinoma (PDAC) is a lethal malignancy, accounting for an estimated 3.2% of new cancer diagnoses in the United States (US).^1^ Pancreatic cancer is expected to account for the third most cancer‐related deaths in 2021.^1^ Many pancreatic adenocarcinomas have already spread beyond the pancreas at the time of diagnosis, with the latest SEER data for 2009–2018 indicating 49.5% of patients have metastatic disease at diagnosis and the 5‐year survival rate for metastatic PDAC (mPDAC) is 3.0%.^2^, ^3^ Approximately half of patients with metastatic pancreatic cancer are able to receive second and subsequent lines of therapy.^4^ PDAC is characterized by a complex immunosuppressive microenvironment, near ubiquity of KRAS mutations—which currently are nontargetable, and treatment paradigms that are dominated by cytotoxic therapies.^5^

In the frontline setting for metastatic disease, the National Comprehensive Cancer Network (NCCN) guidelines recommend either gemcitabine‐based treatment, typically gemcitabine plus nab‐paclitaxel or 5‐fluorouracil (5‐FU) based treatment, often a combination of 5‐FU, oxaliplatin, irinotecan, and folinic acid (FOLFIRINOX).^6^ For patients with tumors driven by mutations in the BRCA pathway and responsive to platinum chemotherapy, the PARP inhibitor olaparib is effective.^7^ For less than 5% of patients, immunotherapy, and targeted therapies, including pembrolizumab, larotrectinib, entrectinib, and olaparib are FDA‐approved or guideline endorsed for small subsets of patients with either microsatellite instability, elevated tumor mutation burden, or a targetable mutation, respectively.^6^, ^8^, ^9^, ^10^ There are limited options for treatment after progression on frontline treatment.

Liposomal irinotecan is a topoisomerase inhibitor indicated for the treatment of patients with mPDAC in combination with fluorouracil and leucovorin (5‐FU/LV), following disease progression on gemcitabine‐based therapy.^11^ The combination of liposomal irinotecan +5‐FU/LV was evaluated in phase 3, open‐label, multicenter NAPOLI‐1 trial. The combination demonstrated significant improvement for overall survival (OS) compared with the control arm of 5‐FU/LV alone, 6.1 months versus 4.2 months (*p* = 0.014), respectively.^12^

It remains unknown as to what extent prior exposure to irinotecan contributes to the likelihood of benefit to liposomal irinotecan.^13^, ^14^ Among the 117 patients treated with liposomal irinotecan +5‐FU/LV in the NAPOL‐1 study, 12 patients (10.2%) were previously exposed to irinotecan. The OS reported for patients previously exposed was 4.6 months compared with 6.7 months for those with no previous exposure.^15^ An earlier analysis of the Flatiron Health data found no association between prior irinotecan exposure and OS among a total of 446 patients treated with liposomal irinotecan.^16^ Results from nonrandomized real‐world studies, with total study sample sizes (with and without prior irinotecan) of 56, 86, and 257 patients treated with liposomal irinotecan, have generated results suggesting prior irinotecan exposure is associated with worse outcomes among patients with mPDAC later treated with liposomal irinotecan.^17^, ^18^, ^19^ A recent real‐world study comparing 86 matched pairs of patients treated with liposomal irinotecan‐based regimens observed no significant differences in survival times and tumor response rates based on prior exposure to irinotecan.^20^

NCCN guidelines currently recommend liposomal irinotecan as Category I unless patients have previously received irinotecan.^6^ As NCCN guidelines can impact payer formulary decisions and prior authorization processes affecting patient access. Thus, studies with larger sample sizes and analyzing other confounding factors are needed to better address this concern, and further analyses may provide greater confidence around these prior conclusions or otherwise.

With the largest dataset to date (*N* = 675) aiming to provide a thorough analysis of this question, this study seeks to understand the impact of prior exposure to irinotecan among patients subsequently treated with liposomal irinotecan in the real‐world setting.

## 2 METHODS

### 2.1 Study design and data source

This retrospective, observational study used the nationwide Flatiron Health electronic health record (EHR) derived, deidentified database. The Flatiron Health database is a longitudinal, demographically, and geographically diverse database from over 280 cancer clinics, comprising deidentified patient‐level structured and unstructured data, curated via technology‐enabled abstraction. The majority of patients in the database originate from community oncology settings; relative community and academic proportions may vary depending on study cohort.^21^ The data are deidentified and subject to obligations, to prevent reidentification and protect patient confidentiality.

### 2.2 Study population

The study population included adult patients diagnosed with mPDAC, who received treatment with a liposomal irinotecan‐containing regimen on or after January 1, 2016, in the metastatic setting. The study period spanned January 2014 until October 31, 2020.

### 2.3 Baseline characteristics

Baseline demographic and clinical characteristics were assessed including age at treatment initiation, disease stage at initial diagnosis, sex, geographic region, race, practice type, and treatment with Whipple procedure. Serum albumin, carbohydrate antigen 19‐9 (CA 19‐9), Eastern Cooperative Oncology Group (ECOG) performance status, and body mass index were assessed within 30 days on or prior to treatment start, prioritizing information that occurred closer to treatment initiation. The number of prior metastatic lines of therapy and exposure to systemic treatments including gemcitabine, 5‐FU, and irinotecan any time prior to treatment initiation was also assessed. Irinotecan exposure in the metastatic setting was defined as receipt of irinotecan any time after 14 days prior to the metastatic diagnosis date, to align with oncologist‐defined, rule‐based lines of therapy in practice, which have been described previously.^19^

### 2.4 Statistical analyses

Baseline demographic and clinical characteristics were analyzed descriptively. Kaplan–Meier methods, with the log‐rank test to determine statistical significance, were used for time‐to‐event analyses (OS, progression‐free survival [PFS]). Real‐world overall survival (rwOS) was defined as the time, in months, from the start of liposomal irinotecan until death (assumed to be the 15th day of the month of death). Patients without a death recorded during the study period were censored on their last visit date.

Real‐world progression‐free survival (rwPFS) was defined as the time, in months, from the start of liposomal irinotecan until the first evidence of either disease progression that occurred more than 14 days after the start of treatment or death any time after treatment initiation. Progression events were abstracted from the clinical notes in the EHR. Abstractors (oncology nurses, advanced practice oncology nurses and tumor registrars) were instructed to identify episodes from which the treating physician concluded there had been tumor growth or worsening of the disease of interest. Notes in which the physician describes disease progression or acknowledges source evidence consistent with progression (e.g., a radiology report) were included in the database. Patients who experienced neither disease progression nor death during the follow‐up period were censored at their last visit date.

Crude and adjusted proportional hazards models were utilized to determine the hazard ratios (HRs) between the irinotecan exposed and irinotecan naïve cohort. The multivariable Cox regression models were adjusted for age at index, ECOG performance score, baseline serum albumin, baseline CA 19‐9, stage at initial diagnosis, race, number of prior lines, prior 5‐FU, prior gemcitabine, prior Whipple surgery, prior progression, and the timing of prior progression. Predictors of disease progression during or following treatment with liposomal irinotecan‐based regimens were assessed based on significance in the multivariable model. *P*‐values less than 0.05 were considered statistically significant. Statistical analyses were performed using SAS statistical software, version 9.4 (SAS Institute Inc., Cary, NC).

## 3 RESULTS

### 3.1 Patient characteristics

**FIGURE 1:**
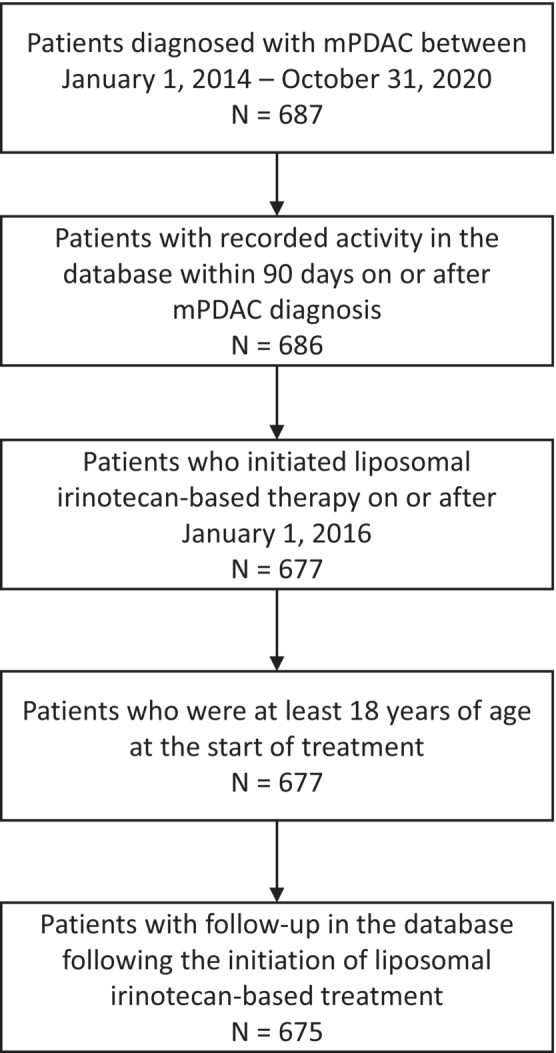
Cohort attrition diagram.

There were 675 patients with mPDAC treated with a liposomal irinotecan‐based regimen during the study period (Figure 1). The median age at treatment initiation was 69 years (IQR: 62–75), and 51.7% of patients were male. One hundred and eighty‐one patients (26.8%) were previously exposed to irinotecan in the metastatic setting, and 240 (35.6%) were exposed at any time prior to treatment initiation (Figure S1). Patient gender and race were similar between those previously treated with irinotecan. Patients who were not previously treated with irinotecan were older than those who had prior exposure. Patients with prior irinotecan treatment were more likely to be diagnosed with de novo mPDAC (71.3% vs. 48.4%). ECOG performance scores were similar between cohorts based on prior treatment. A majority of patients with prior exposure to irinotecan had experienced two or more prior lines of therapy (84.5%). Five hundred and forty patients (80.0%) previously experienced disease progression event before initiating liposomal irinotecan‐based treatment. Baseline characteristics are described in Table 1.

**TABLE 1:** Patient demographic and clinical characteristics.

	Overall	Prior irinotecan exposure in metastatic setting	No prior irinotecan exposure in metastatic setting
Total	675 (100.0%)	181 (100.0%)	494 (100.0%)
Age at index			
65 and younger	245 (36.3%)	91 (50.28%)	154 (31.17%)
66 and older	430 (63.7%)	90 (49.72%)	340 (68.83%)
Mean (SD)	68.2 (9)	64.8 (8.4)	69.5 (8.9)
Median (Q1–Q3)	69 (62–75)	65 (59–71)	71 (63–76)
Sex			
Male	349 (51.7%)	94 (51.93%)	255 (51.62%)
Female	326 (48.3%)	87 (48.07%)	239 (48.38%)
Stage at initial diagnosis			
Stage IV	368 (54.52%)	129 (71.27%)	239 (48.38%)
Other	307 (45.48%)	52 (28.73%)	255 (51.62%)
Race			
Asian	15 (2.22%)	3 (1.66%)	12 (2.43%)
White	484 (71.7%)	140 (77.35%)	344 (69.64%)
Other Race/missing	176 (26.07%)	38 (20.99%)	138 (27.94%)
Geographic region			
Northeast	110 (16.3%)	39 (21.55%)	71 (14.37%)
Midwest	97 (14.37%)	22 (12.15%)	75 (15.18%)
South	296 (43.85%)	84 (46.41%)	212 (42.91%)
West	106 (15.7%)	19 (10.5%)	87 (17.61%)
Unknown	63 (9.33%)	17 (9.39%)	46 (9.31%)
ECOG score			
0	118 (17.48%)	35 (19.34%)	83 (16.8%)
1	276 (40.89%)	71 (39.23%)	205 (41.5%)
2+	115 (17.04%)	28 (15.47%)	87 (17.61%)
Missing	166 (24.59%)	47 (25.97%)	119 (24.09%)
Practice type			
Academic	57 (8.44%)	14 (7.73%)	43 (8.7%)
Community	618 (91.56%)	167 (92.27%)	451 (91.3%)
Previous lines of therapy			
0	101 (14.96%)	0 (0.0%)	101 (20.45%)
1	319 (47.26%)	28 (15.47%)	291 (58.91%)
2 or more	255 (37.78%)	153 (84.53%)	102 (20.65%)
Prior irinotecan (metastatic setting)			
Yes	181 (26.81%)	181 (100.0%)	0 (0.0%)
No	494 (73.19%)	0 (0.0%)	494 (100.0%)
Prior irinotecan (any setting)			
Yes	240 (35.56%)	181 (100.0%)	59 (11.94%)
No	435 (64.44%)	0 (0.0%)	435 (88.06%)
Prior progression			
Yes	540 (80.0%)	170 (93.92%)	370 (74.9%)
No	135 (20.0%)	11 (6.08%)	124 (25.1%)

### 3.2 Clinical outcomes

#### 3.2.1 Overall survival—crude/unadjusted

**FIGURE 2:**
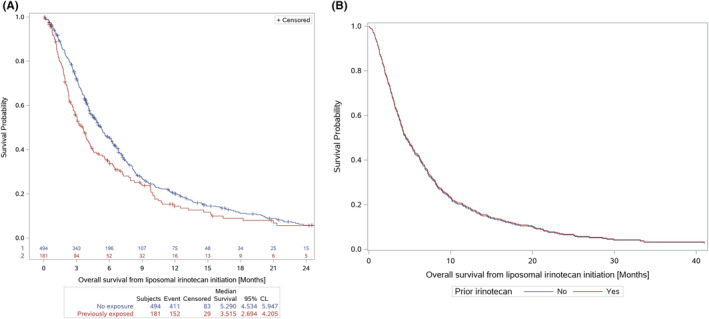
Unadjusted (A) and direct‐adjusted (B) real‐world overall survival by exposure to prior irinotecan in the metastatic setting. Adjustment for covariates mitigates clinical impact of prior irinotecan exposure (distance between KM curves is reduced).

The overall median rwOS was 4.7 months (95% CI: 4.2–5.3 months). rwOS was longer for patients treated with liposomal irinotecan in earlier lines of therapy, first line (1L): 6.9 months (5.3–8.3 months); second line (2L): 5.0 months (4.2–6.3 months); and third line plus (3L+): 3.8 months (3.2–8.3 months). Patients who were previously exposed to irinotecan experienced worse survival compared with those who were not exposed in the metastatic setting: 3.5 months (2.7–4.2 months) versus 5.3 months (4.5–6.1 months) (Figure 2A). Similar results were also observed for those exposed to irinotecan at any time prior (Figure S**2**). Unadjusted, the HR comparing rwOS based on exposure to prior irinotecan was 1.29 (95% CI: 1.07–1.56), *p* < 0.05. The rwOS results are presented in Table 2.

**TABLE 2:** Unadjusted real‐world overall survival.

Category	*N*	Deaths	Median OS (95% CI)
Overall	675	563	4.7 (4.2–5.3)
Line group			
1	101	73	6.9 (5.3–8.3)
2	319	271	5 (4.2–6.3)
3+	255	219	3.8 (3.2–4.3)
Prior irinotecan in metastatic setting			
Yes	181	152	3.5 (2.7–4.2)
No	494	411	5.3 (4.5–5.9)
Prior irinotecan in any setting			
Yes	240	201	3.8 (3.1–4.5)
No	435	362	5.3 (4.5–6.1)
Timing of irinotecan administration prior to index			
0–3 months prior	51	42	3.2 (2.3–5.8)
3–6 months prior	48	42	2.1 (1.4–3.5)
6–12 months prior	60	52	3.7 (2.7–4.6)
>12 months prior	22	16	9.8 (5.1–16.4)

#### 3.2.2 Progression‐free survival—crude/unadjusted

**FIGURE 3:**
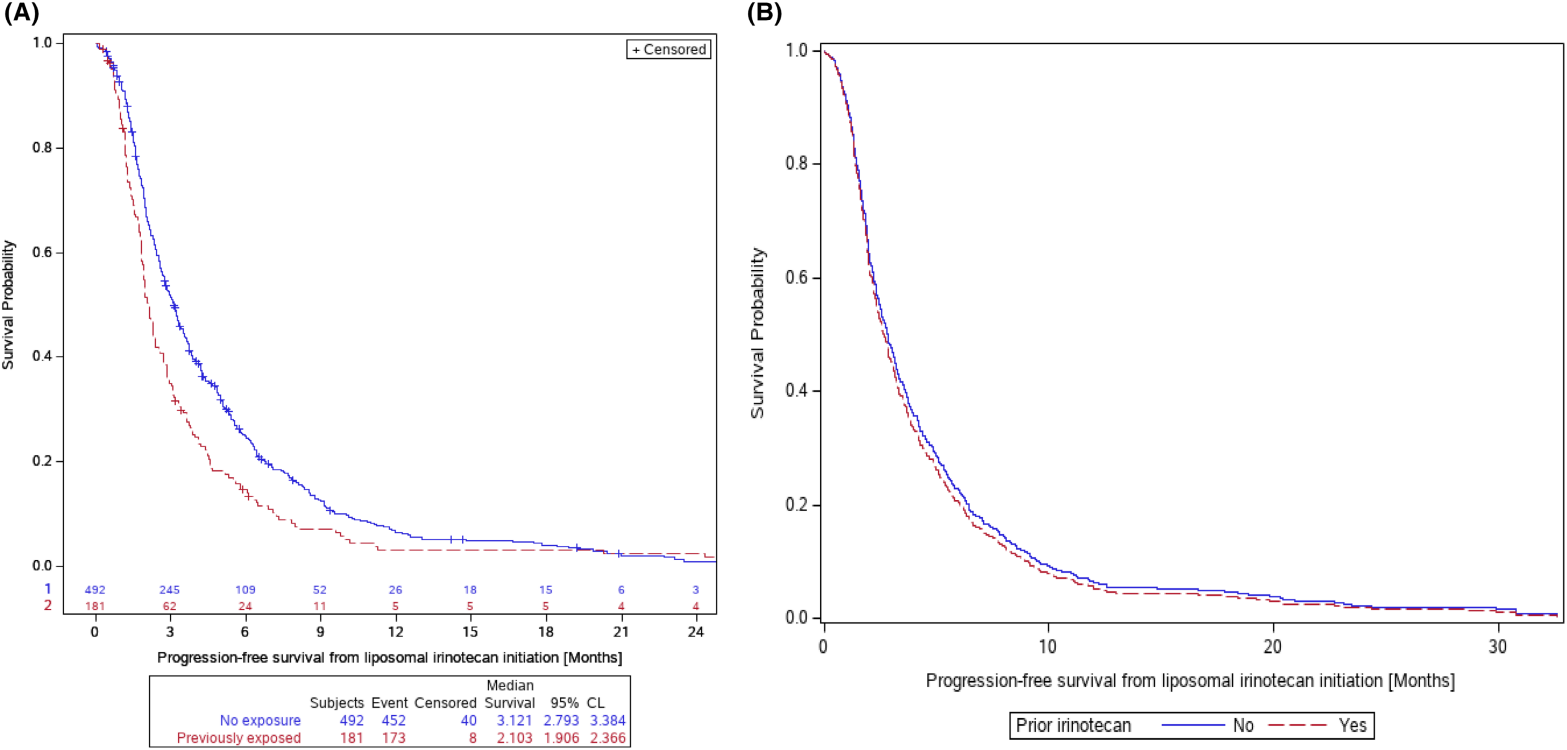
Unadjusted (A) and direct‐adjusted (B) real‐world progression‐free survival by exposure to prior irinotecan in the metastatic setting. Adjustment for covariates mitigates clinical impact of prior irinotecan exposure (distance between KM curves is reduced).

The overall median rwPFS was 2.8 months (95% CI: 2.5–3.1). rwPFS was longer for patients treated with liposomal irinotecan in earlier lines of therapy, 1L: 3.8 months (2.9–4.8 months); 2L: 3.2 months (2.8–3.5 months); and 3L+: 2.1 months (1.9–2.3 months). Patients who were previously exposed to irinotecan experienced worse rwPFS compared with those who were not exposed in the metastatic setting: 2.1 months (1.9–2.4 months) versus 3.1 months (2.8–3.5 months) (Figure 3A). Similar results were observed for those exposed at any time prior (Figure S3). Unadjusted, the HR comparing rwPFS based on exposure to prior irinotecan was 1.40 (95% CI: 1.18–1.67), *p* < 0.05. The rwPFS are presented in Table S1.

#### 3.2.3 Predictors of progression

A number of variables were significant predictors of disease progression during or following treatment with liposomal irinotecan‐based regimens in univariable models, including prior irinotecan exposure, age at index, ECOG performance score, baseline serum albumin, stage at initial diagnosis, number of prior lines of therapy, and prior disease progression. In the multivariable model age at index, ECOG performance score, prior 5‐FU exposure, and prior disease progression remained significant predictors, adjusted for other baseline variables. The covariates identified as significant predictors of progression during, or following, treatment with liposomal irinotecan are summarized in Table S2. ECOG PS was identified as the strongest predictor of progression in the multivariable model followed by prior disease progression and age at treatment initiation.

#### 3.2.4 Adjusted overall survival and progression‐free survival

**FIGURE 4:**
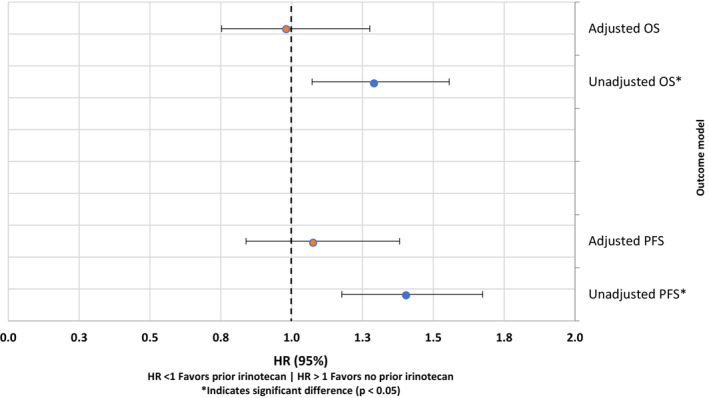
Unadjusted and adjusted outcomes models forest plot. Adjusted for: age at index, ECOG Performance score, Baseline serum albumin, baseline CA 19‐9, stage at initial diagnosis, race, number of prior lines, prior 5‐FU, prior Gemcitabine, Prior Whipple, prior progression and the timing of prior progression. PFS, progression free survival; OS, overall survival; HR, Hazard ratio. *All unadjusted models significant, *p* < 0.05.

After adjustment for demographic and clinical characteristics, patients who were previously exposed to irinotecan experienced a similar risk of mortality relative to patients who were not previously exposed (adjusted rwOS HR: 0.98; 95% CI: 0.75–1.28, *p* = 0.8836). No significant difference was observed in the adjusted risk of disease progression based on prior exposure to irinotecan in the metastatic setting (adjusted rwPFS HR: 1.08; 95% CI: 0.84–1.38, *p* = 0.5626). The summary of the adjusted models is presented in Figure 4. The estimated median adjusted rwOS was 4.5 months for patients both with and without prior exposure to irinotecan (Figure 2B). The estimated median adjusted rwPFS among patients with prior exposure to irinotecan was 2.6 months versus 2.8 months for patients who were not previously exposed (Figure 3B).

## 4 DISCUSSION

To our knowledge, this is the largest real‐world study, to date, of patients with mPDAC treated with liposomal irinotecan‐based regimens. The results suggest prior exposure to irinotecan did not preclude clinical benefit for overall and PFS compared with irinotecan‐naïve patients. PFS observed in our study was similar to that observed in the NAPOLI‐1 trial (3.1 months vs. 2.8 months) despite the fact that our cohort of real‐world patients with mPDAC were more pretreated, older, and had worse performance status than the cohort of patients included the clinical trial.^12^

The overall rwOS and rwPFS at 4.7 months and 2.8 months in our study, respectively, were consistent with those reported in our single academic center study among patients treated with liposomal irinotecan, 5.3 months and 2.9 months, respectively.^17^ In the single‐center study, we also noted that the unadjusted median OS and PFS were worse for patients who had previously been exposed to irinotecan and experienced progression on prior irinotecan‐based regimen, 3.9 months and 2.2 months, respectively, compared with the unexposed, 7.7 months and 4.6 months, respectively.^17^ A real‐world study which also utilized the Flatiron Health database reported rwOS 4.1 months (95% CI: 3.0–5.3) among patients who had previously received irinotecan compared with 5.6 months (95% CI: 4.7–6.9) for the unexposed population.^19^ Likely due to the sample sizes of the aforementioned studies, no multivariable analyses were performed to assess the role of other clinical characteristics that could impact the relationship between prior exposure to irinotecan and clinical outcomes.

The subgroup analyses of the NAPOLI‐1 trial also reported prior irinotecan were independently associated with patient outcomes.^16^ Results from our study were similar to those reported on the subgroup trial population. There were no multivariable analyses performed on the clinical trial data. A nomogram was developed to predict survival from NAPOLI‐1 cohort.^22^ Among the 417 patients included in the trial, prior irinotecan was not significantly associated with OS in the univariate analysis and was not included in the final nomogram. Performance score, baseline albumin, liver metastases, and stage were key predictors among the variables analyzed.^22^ In a separate multivariable model constructed to assess OS of patients with advanced PDAC in the second‐line setting, prior therapy did not appear in the final model.^23^

Among the 86 patients treated with liposomal irinotecan in the Korean Cancer Study Group, analysis by overall and PFS was better than what we report for our cohort.^18^ This is consistent with the robust clinical outcomes experienced by Asian patients with mPDAC who are treated with liposomal irinotecan‐based regimens.^18^, ^24^, ^25^, ^26^ Despite this, Yoo et al. still reported that patients who received FOLFIRINOX prior to liposomal irinotecan in their study experienced significantly reduced PFS relative to gemcitabine‐based therapy; no significant difference was observed for OS. Similar outcomes to our study were reported among 58 patients with mPDAC from two medical centers in the US treated with liposomal irinotecan +5‐FU/LV, which did not find an association with prior irinotecan and OS.^27^

Adding to the evidence of prior studies, we found that among patients treated with prior irinotecan in our study, time from prior irinotecan exposure was a significant predictor of outcomes with patients more recently exposed experiencing less to no benefit relative to those with a longer time interval since prior exposure. In particular, response to liposomal irinotecan‐based regimens was enriched among patients who were previously exposed to irinotecan more than 90–120 days prior. However, those with a longer time from prior irinotecan may also be fitter patients who have survived long enough to receive additional treatments. In a sensitivity analysis, time from prior gemcitabine was assessed with similar results observed, that is, longer time from prior gemcitabine was associated with improved outcomes. This additional sensitivity analysis suggests that, independent of prior treatment, patients who survive an extended period of time before receiving additional therapies are likely to experience better outcomes than those who have a short time between treatment.

## 5 LIMITATIONS

The limitations of these analyses are important. Data from the Flatiron Health database are collected prospectively for routine clinical care, and in this study, these data were evaluated retrospectively for research purposes. Patient data included in the analysis were subject to nonrandom allocation bias and the reasons to forgo treatment are not available from the database. Thus, there were few patients who received liposomal irinotecan immediately following FOLFIRINOX in our cohort and their outcomes could not be evaluated robustly. Additionally, too few patients received a liposomal irinotecan‐based regimen soon after irinotecan exposure (e.g., 30–60 days after) for a robust evaluation of their clinical outcomes. The Flatiron Health EHR data are sourced primarily from the community setting and therefore may not be generalizable to all oncology practices. The max age reported in the database is 85 years, which may impact generalizability of our results for older adults. Real‐world imaging to assess disease progression may occur less frequently than clinical trials, limiting the comparison of real‐world progression to clinical trials. Also, the determination of progressive disease may be derived from purely clinical assessment or based on clinical proxies such as serum CA 19‐9 and may not align with formal RECIST criteria. There are additional confounding factors and biases that are not well captured in these data including the sites of metastases, additional comorbidity burden, receipt of acute care or hospitalization, which may have impacted the clinical outcomes observed in our study.

## 6 CONCLUSIONS

This is the largest study to date reporting the impact of prior irinotecan in patients subsequently treated with liposomal irinotecan. Prior irinotecan was not found to be a significant predictor of patient outcomes in those subsequently treated with liposomal irinotecan. Thus, the results may inform the rationale for utilizing liposomal irinotecan combination therapy following prior irinotecan exposure in mPDAC, in particular where the prior irinotecan exposure was more distant in time.

## AUTHOR CONTRIBUTIONS

**Kenneth H. Yu:** Conceptualization (equal); investigation (equal); methodology (equal); validation (equal); writing – review and editing (equal). **Paul Cockrum:** Conceptualization (equal); funding acquisition (equal); investigation (equal); methodology (equal); project administration (equal); supervision (equal); writing – original draft (equal); writing – review and editing (equal). **Andy Surinach:** Conceptualization (equal); formal analysis (equal); investigation (equal); methodology (equal); project administration (equal); validation (equal); visualization (equal); writing – review and editing (equal). **Neil Lamarre:** Formal analysis (equal); investigation (equal); validation (equal); visualization (equal); writing – review and editing (equal). **Shu Wang:** Formal analysis (equal); investigation (equal); validation (equal); visualization (equal); writing – review and editing (equal). **Eileen M. O'Reilly:** Conceptualization (equal); investigation (equal); methodology (equal); validation (equal); writing – review and editing (equal).

## FUNDING INFORMATION

This study was sponsored by Ipsen. The sponsor was involved in the design of the study, analysis, and interpretation as well as final approval of the manuscript.

## CONFLICT OF INTEREST STATEMENT

K.Y. serves in an advisory role for Ipsen and receives research funding from Halozyme, Bristol‐Myers Squibb, and Ipsen.

P.C. is an employee of and has stock in Ipsen.

A.S., S.W., and N.L. are employees of Genesis Research, which receives consulting fees from Ipsen.

E.O. serves in an advisory role for Cytomx Therapeutics (DSMB), Rafael Therapeutics (DSMB), Seagen, Boehringer Ingelheim, BioNTech, Ipsen, Merck, IDEAYA, AstraZeneca, Noxxon, BioSapien, Cend Therapeutics, Thetis; her employer receives research funding from Genentech/Roche, Celgene/BMS, BioNTech, AstraZeneca, Arcus, Elicio, Parker Institute, and she and has an immediate family member who serves in consulting role for Agios, Genentech/Roche, Eisai.

## ETHICS STATEMENT

The data accessed were deidentified in accordance with the HIPAA Privacy Rule, and no personal health information was extracted. Therefore, the study did not require informed consent or institutional review board approval.

## PATIENT CONSENT

Informed consent was waived as this was a noninterventional study using routinely collected data. The data are deidentified and subject to obligations to prevent reidentification and protect patient confidentiality.

## Supporting information


**Supporting information S1.** Supplementary material

## Data Availability

The data that support the findings of this study have been originated by Flatiron Health, Inc. These de‐identified data may be made available upon request, and are subject to a license agreement with Flatiron Health; interested researchers should contact DataAccess@flatiron.com> to determine licensing terms.
