# Supplementary material for: Prior irinotecan exposure does not preclude benefit to liposomal irinotecan in patients with metastatic pancreatic ductal adenocarcinoma
Source: Cancer Med. 2023 Mar 19;12(8):9496–505. doi: 10.1002/cam4.5714 (PMC10166959; doi:10.1002/cam4.5714)
Supplement: Supplementary file 1 — Supporting information S1. Supplementary material [file CAM4-12-9496-s001.docx]

**Supplemental Figure 1 – Distribution of prior irinotecan exposure in the metastatic (A) and in any (B) setting**

**Supplemental Figure 2 – Unadjusted real-world overall survival by exposure to prior irinotecan in any setting**

**
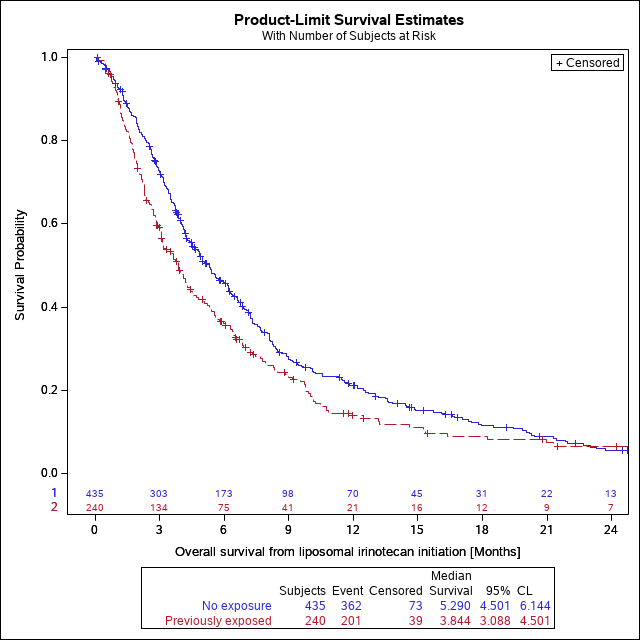
**

**Supplemental Figure 3 – Unadjusted real-world progression-free survival by exposure to prior irinotecan in the metastatic setting**

**
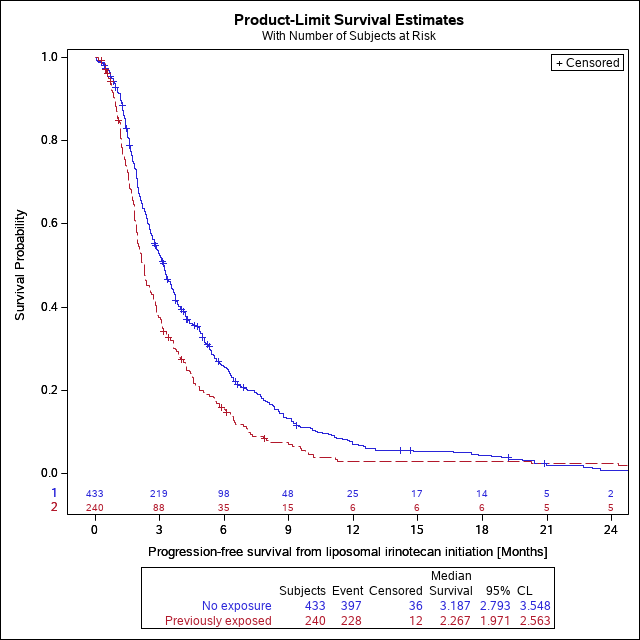
**

**Supplemental Table 1: Unadjusted Real-world progression-free survival (PFS)**

| **Category** | **N** | **Events** | **Median PFS (95%CI)** |
| --- | --- | --- | --- |
| Overall | 673 | 625 | 2.8 (2.5 - 3.1) |
| **Line Group** |  |  |  |
| 1 | 101 | 87 | 3.8 (2.9 - 4.8) |
| 2 | 318 | 298 | 3.2 (2.8 - 3.5) |
| 3+ | 254 | 240 | 2.1 (1.9 - 2.3) |
| **Prior irinotecan in metastatic setting** | | |  |
| Yes | 181 | 173 | 2.1 (1.9 - 2.4) |
| No | 492 | 452 | 3.1 (2.8 - 3.4) |
| **Prior irinotecan in any setting** | |  |  |
| Yes | 240 | 228 | 2.3 (2 - 2.6) |
| No | 433 | 397 | 3.2 (2.8 - 3.5) |
| **Timing of irinotecan administration prior to index** | | | |
| 0 to 3 months prior | 51 | 48 | 1.9 (1.7 - 2.7) |
| 3 to 6 months prior | 48 | 47 | 1.8 (1.2 - 1.9) |
| 6 to 12 months prior | 60 | 56 | 2.3 (2 - 3.1) |
| > 12 months prior | 22 | 22 | 3.5 (1.5 - 6.1) |

**Supplemental Table 2: Predictors of real-world progression**

| **Variable** | **p-value** | **p-value** |
| --- | --- | --- |
|  | **(in univariable model)** | **(in multivariable model)** |
| Prior Irinotecan (metastatic setting) | 0.0002 | 0.5626 |
| Prior Irinotecan (any setting) | 0.0002 | 0.8831 |
| Age at index | 0.0018 | 0.0253 |
| ECOG Score | <.0001 | <.0001 |
| Serum albumin | 0.0344 | 0.2239 |
| Race | 0.4938 | 0.3852 |
| Site of tumor | 0.0989 | 0.4049 |
| CA 19-9 | 0.0616 | 0.2257 |
| Sex | 0.464 | 0.5548 |
| Stage | 0.0058 | 0.1956 |
| Prior Lines of Therapy | <.0001 | 0.1686 |
| Prior fluorouracil | <.0001 | 0.0492 |
| Prior gemcitabine | 0.7158 | 0.7203 |
| Prior Whipple | 0.8159 | 0.7393 |
| Prior disease progression | <.0001 | 0.0023 |
| Timing of prior disease progression | <.0001 | 0.8807 |

**Supplemental Table 3 -** **Cox progression hazard models**

|  |  | **Unadjusted Results*** | | | **Adjusted Results** | | | |
| --- | --- | --- | --- | --- | --- | --- | --- | --- |
| **Outcome** | **Variable** | **HR** | **Lower 95%** | **Upper 95%** | **HR** | **Lower 95%** | **Upper 95%** | **p-value** |
| PFS | Prior Irinotecan (metastatic setting) | 1.40 | 1.18 | 1.67 | 1.08 | 0.84 | 1.38 | 0.5626 |
| PFS | Prior Irinotecan (any time) | 1.37 | 1.16 | 1.61 | 1.02 | 0.79 | 1.32 | 0.8831 |
| *PFS*** | *Prior Irinotecan (metastatic setting)* | *1.40* | *1.18* | *1.67* | *1.09* | *0.86* | *1.39* | *0.4732* |
| *PFS*** | *Prior Irinotecan (any time)* | *1.37* | *1.16* | *1.61* | *1.03* | *0.79* | *1.33* | *0.8544* |
| OS | Prior Irinotecan (metastatic setting) | 1.29 | 1.07 | 1.56 | 0.98 | 0.75 | 1.28 | 0.8836 |
| OS | Prior Irinotecan (any time) | 1.25 | 1.05 | 1.49 | 0.98 | 0.75 | 1.28 | 0.8742 |
| *OS*** | *Prior Irinotecan (metastatic setting)* | *1.29* | *1.07* | *1.56* | *1.00* | *0.78* | *1.29* | *0.9811* |
| *OS*** | *Prior Irinotecan (any time)* | *1.25* | *1.05* | *1.49* | *0.98* | *0.75* | *1.29* | *0.8959* |

Adjusted for: age at index, ECOG Performance score, Baseline serum albumin, baseline CA 19-9, stage at initial diagnosis, race, number of prior lines, prior 5-FU, prior Gemcitabine, Prior Whipple, prior progression and the timing of prior progression

PFS: progression free survival, OS: overall survival

*All unadjusted models significant, p < 0.05

**Excluding Prior Line of Therapy from the model
